# The Italian Surgical Society

**Published:** 1886-07

**Authors:** A. Lagorio

**Affiliations:** Chiavari, Italy


### FOREIGN CORRESPONDENCE.

*To the Editors of the* CHICAGO MEDICAL JOURNAL AND EXAMINER,

*Gentlemen:*

The third annual meeting of the Italian Surgical Society was held in Rome on the 19th, 20th and 21st of April. President Durante occupied the chair, and after an appropriate address opened the meeting for work.

Professor Trombetta, of Messina, read the first paper on the total extirpation of the thyroid gland, and on the strumous cachexia which may occur after the operation, as was first called to our attention by Tuillard and Köcher. He had performed this operation over a year ago, and the patient is now enjoying good health, showing no tendency to the cachexia. He cited the results of Köcher, Baumgarten, Credè, Mikulicz, in 55 operations, of which only 15 (27 per centum) became cachectic, while 40 (73 per centum) showed no tendency to it.

Professor Caselli, of Genoa, had performed five extirpations; the first seven years ago, and the last two years ago. The patients are at present in the best of health, except the third case, which died three days after the operation. Professor Ruggi also reported five operations. Two ended in cachexia, and three are well. Professors Ceccherelli and D'Antona each reported three cases.

Dr. Granati reported a very strange case of laparotomy and caesarian operation, which, if it were not for the well-known truthfulness of the doctor, and the fact of its being vouched for by equally well-known physicians, would seem incredible. A country-woman, 23 years old, and unmarried, became pregnant. Being almost at full term, and wishing to conceal her condition, she attempted to produce a miscarriage upon herself by passing a long knitting needle into the vagina so as to puncture the amniotic sac. She had punctured four or five times the anterior lip of the neck of the womb, but with negative results. Wishing to avoid a medical examination, which was threatened by her employers, on the 28th of last March, she took a large kitchen knife, and with it opened her abdomen on the right side, making an oblique wound from within outwards and from below upwards of about 12 centm. in length. She also incised the uterus in the same manner and attempted to draw out the fœtus. But th s being too large to pass through the wound, she severed an arm, and the head from the trunk, so as to reduce it in volume, and by so doing she was enabled to extract the whole fœtus. Having emptied the uterus also by taking out the placenta, she put a large bandage around her belly, strongly tightened, got up from bed, and hid the fœtus in the mattress. Then she dressed herself, did some housework, and went to the nearest town (Viterbo) on a wagon. She visited one of her sisters, to let her know that she could not be pregnant, for her underclothes were still wet with menstrual blood. After having stopped here about two hours, she returned to her home in the country, walking about a mile, but when she reached the house she was taken with a severe vomiting and fainted. Drs. Serpieri and Baliva were sent for by her parents. They found a wound as described above, and a mass of small intestines protruding. They replaced these, and dressed the wound as the case required.

Not the least sign of a peritonitis has ensued, excepting a slight tenderness around the wound. There has been no vomiting; no hiccough; very little thirst; the tongue has been rosy and moist, and the alvine discharges normal. The highest temperature has been 38½° C. (101° F.); the pulse never above hundred. The patient has always slept well and eaten well. No complications have arisen in the uterus and its appendages. The lochial discharges have been normal. At present, 21 days after the wound was inflicted, the abdominal wound still suppurates and is not yet closed. She constantly denied any accomplices, and insisted that the punctures in the uterus and the laparotomy have been done exclusively by herself. This case is really extraordinary.

Ruggi reported several cases operated on for tumors on the head and neck. Caselli desired to know if the members ever observed any cardiac or respiratory disturbances following a stretching, displacement or lesion of any kind of the pneumogastric nerve. He had lost two cases in over a hundred operations, in which he had been obliged to stretch or displace the nerve. He favored the incision of the nerve, rather than irritating it. Ferrari had seen this procedure done by Volkmann. He thought the cause to be in the chemical irritation of the antiseptic used in the operation. Durante recalled an operation for the removal of a sarcoma of the neck, during which he had been obliged to dissect, stretch and displace the nerve. A month after the patient began to complain of some disturbance of respiration.

Durante read an elaborate paper on his new process of resection of the knee joint. His operation consists in removing the bony tissue from the two articular extremities of the femur and tibia in such a manner that the wedge formed of the tibia enters an analogous space made in the femur. By so doing we obtain an exact approximation of the bony surfaces without any danger of lateral displacement. To avoid any antero-posterior displacement, he preserves the patella, by taking off only the cartilaginous layer and fixing it by means of a Thiersch nail to the head of the tibia. He has had good results in four operations.

Triconi read a paper on suppuration due to micro-organisms. His results do not differ from those of Rossbach, Garré, etc., with the exception that he has found in abscesses and boils only one staphylococcus, which he claims to be specific. It was not found in the blood and does not produce osteomyelitis.

Following this paper, the same author read another, on senile gangrene. He drew the following conclusions: 1. Senile gangrene is due to a small bacillus, short and thin, with rounded extremities, with a spore in the center or in the sides. 2. This bacillus circulates in the blood of patients affected with senile gangrene (has seen it three times). 3. It exists in the gangrenous icor, in the demarcation line, in the blood, in the heart of the cadaver, and in the lymphatic spaces of the skin and subcutaneous tissue. 4. Experiments of culture can be made in gelatine, in beef tea, in agaragar, in blood serum, and potatoes. 5. Colors well with aniline, especially red and violet. He has transmitted the disease to calves, rabbits, and rats.

Ceci reported a case of rupture of the tendon of the patella at its insertion in the tibia. He sutured it and had a good result.

Lampugnani reported 166 successful orthopedic operations of the bones.

Margary sent a paper on the treatment of flat foot, by the extirpation of the astragalus.

D'Antona read a very interesting paper on renal surgery, reporting seven nephrectomies.

Caselli reported three cases of laparotomy. The last for the treatment of a purulent peritonitis. He washed the abdominal cavity every two hours for the first two days with an antiseptic solution, and had good success. This is the first case of the kind ever treated in Italy in such a manner.

Ceci impressed the SOCIETY by presenting a successful extirpation of an enlarged spleen. The patient was a female, only 17 years old, and not well developed. The operation was performed on the 20th of last March, in the Pammatone Hospital at Genoa. The bichloride of methylene was first used as an anæsthetic, but the narcosis was slow and irregular. An incision 23 centm. long was made in the *linea alba*, but during the hæmostasia of the abdominal walls, symptoms of suffocation supervened, and the operation was interrupted for half an hour. Chloroform was then substituted for the bichloride of methylene with good effect. The enlarged spleen was drawn through the wound, and the peduncle, 13 centm. large, was ligated with three sutures. Very little blood was lost, and the operation lasted one hour and a half. The extracted spleen weighed 2,400 grams, or 1–16th the weight of the body of the patient. A furious delirium followed and lasted until the next morning. After this there appeared a rapidly progressive shock, and the temperature went down to 35.8° C. (96.5° Fah.).

This danger having been overcome, the pulse became very rapid, with impossibility of counting it, the respiration

60 to 80. This condition lasted two days. Great benefit was derived from oxygen inhalations and from nourishing and stimulating food given by enemas. The respiration fell to 30 and the pulse 120. On the fourth day fever appeared. On the eighth the dressing was changed. Suppuration was found in the line of the sutures, and purulent peritonitis. This septic process was immediately aborted by energetic disinfection. Erysipelas of the abdominal walls then followed, and was also overcome. On the 16th day Professor Ceci declared the patient out of danger, and at present (a month after) is almost well. This is the first operation of the kind attempted in Genoa, and the second in Italy with a recovery, the fortieth on record, and the eighth with a cure.

Durante related two cases in which he had made an artificial anus, as a preparatory treatment in severe diseases of the rectum.

Ceccherelli reported a case of laparotomy for a fœtal inclusion. The patient was a girl 11 years old, who for years had suffered pains in the abdomen and had noticed a tumor on the left side. A diagnosis was made of a multilocular ovarian cyst, with calcareous patches in the walls of the cyst. Ovariotomy was performed, and in 15 days the patient was well. On examining the tumor it was found to be a fœtal inclusion, and precisely of a type known as *engastro-amorphus*.

Mugnai reported a number of experiments made in the surgical clinique of Rome, on local anæsthesia produced by hypodermic injections of a 5 per centum solution of cocaine. He had been able to do several operations such as external urethrotomy, extirpation of a sarcoma of the inguinal region, two removals of testicles, etc. Professor Caselli confirmed these results, and believed that with cocaine we can do two-thirds of surgery without using the usual anæsthetics.

Lampiasi reported two cases of ligature of the subclavian artery.

Postempski presented two cases of echinococcus of the liver treated with the Recamier caustic. In both cases he had hernia of the abdominal viscera. In one, a protrusion of the stomach, and in the other of the small intestines.

Mazzoni read a paper on intestinal resection for gangrenous herniæ. He had a successful result in one case. The statistics show that in Italy, up to 1884, the results have not been good. Mazznechelli had three deaths in four operations. But lately the results have been better. In 15 operations only five died, three from preexisting peritonitis, and two from other causes. He advised silk or catgut for sutures. The surgeon should have no fear of resecting as much tissue as necessary, for it is imperative to reach the sound tissue.

Torretta spoke on hip-joint disease. He believes in early resections, for he claims they will give better results than the palliative or expectant treatment.

Other papers of little importance were read and discussed, after which the committee on nomination presented the following list of names, to act as officers for the ensuing year:

A. Caselli, president; A. D'Antona, A. Ceci, vice-presidents; Caneva, secretary.

The retiring president, after having reviewed the scientific work of the meeting, thanked the members of the society for their papers and for the interest and harmony shown in the discussions; hailed at Genoa as the next place of meeting, and amid a general applause declared the third meeting of the Italian Surgical Society adjourned.

A. LAGORIO.

Chiavari, Italy, May, 1886.

